# Gardeniae Fructus Enhances Skin Barrier Function via AHR-Mediated FLG/LOR/IVL Expression

**DOI:** 10.3390/molecules30183764

**Published:** 2025-09-16

**Authors:** Kaile Zong, Fangni Zhou, Kewei Xu, Junzi Dong, Qing Huang, Jianxin Wu

**Affiliations:** 1 Skin Health and Cosmetic Development & Evaluation Laboratory, China Pharmaceutical University, Nanjing 211198, China; 3320021289@stu.cpu.edu.cn (K.Z.); 3322020930@stu.cpu.edu.cn (K.X.); huangqing@cpu.edu.cn (Q.H.); 2 Efficacy Skin Care Research and Development Center, Shanghai Junyu Biotechnology Group Co., Ltd., Shanghai 200050, China; zhoufangni@juny.cn (F.Z.); djz@juny.cn (J.D.)

**Keywords:** Gardeniae Fructus, natural products, skin protection, extracts derived from plants, skin barrier function

## Abstract

Gardeniae Fructus (GF), a traditional Chinese medicine rich in iridoids, has demonstrated skin-improving effects. However, its mechanisms for enhancing epidermal barrier function remain unclear. In this study, the iridoids in GF were characterized using UPLC-MS/MS. The improvement in the barrier function by GF was assessed through in vitro experiments and a human efficacy assessment. In addition, the potential targets were predicted through proteomics analysis, molecular docking, and molecular dynamics (MD), and verified in HaCaT cells and three-dimensional epidermal models. Nine iridoids were identified in GF. In vitro, GF effectively promoted cell migration and reduced cell damage and oxidative stress. Proteomics analysis combined with molecular docking and MD simulations predicted that the primary iridoids in GF ameliorate barrier function by binding to the aryl hydrocarbon receptor (AHR) with high affinity and stability. Subsequent validation demonstrated that GF significantly upregulated AHR, filaggrin (FLG), loricrin (LOR), and involucrin (IVL) mRNA and protein expression. A 28-day randomized double-blind human efficacy assessment in subjects with sensitive skin showed that the gel with GF increased stratum corneum hydration, reduced transepidermal water loss (TEWL), and lowered erythema index and lactic acid tingling. These findings suggest that GF enhances the skin barrier via AHR activation-mediated upregulation of barrier proteins, supporting its cosmeceutical potential.

## 1. Introduction

The human skin serves not only as a symbol of appearance but also as a vital barrier against the external environment, reflecting an individual’s general age and health status [1,2]. Maintaining a stable skin barrier is fundamental to skin health, as it participates in regulating the transport of water, and limits the invasion of microbes, allergens, and irritants [3,4]. Factors such as excessive stress, unhealthy habits, and air pollution can compromise the skin barrier, increasing susceptibility to skin diseases [5,6,7,8]. Approximately 25% of the population suffers from skin diseases like acne vulgaris, psoriasis, and atopic dermatitis in today’s society [9], while around 37% of people are troubled by skin sensitivity [10]. Affected individuals often exhibit reduced skin hydration, heightened reactivity, and increased sensitivity [11]. Such compromised skin negatively impacts both physical and mental well-being, significantly diminishing quality of life. Thus, cosmetic products offering soothing and barrier-repairing effects have become a main market demand. The active substances extracted from natural plants represent a promising approach increasingly welcomed and expected by consumers.

In recent years, traditional Chinese medicines (TCMs) have been widely used in cosmetic raw materials due to their excellent efficacy and high safety [12]. Gardeniae Fructus (GF), the dried and ripe fruit of *Gardenia jasminoides* Ellis, was first recorded in the ancient Chinese text “Shennong Materia Medica” and has a long history of dual use as both medicine and food. GF has been employed in skincare since the Tang Dynasty for its properties in clearing heat, reducing swelling, and cooling blood. Iridoids constitute the primary active components of GF [13]. Studies have demonstrated that iridoids from GF could exert significant anti-inflammatory effects, scavenge reactive oxygen species (ROS), and activate the Nrf2 pathway [14,15]. Furthermore, iridoids have shown effects in promoting wound healing and improving skin lesions [16,17]. The aryl hydrocarbon receptor (AHR) is a ligand-activated transcription factor found in the cytoplasm, responsive to diverse exogenous and endogenous ligands, which elicits varied biological effects [18]. Research indicates that antioxidant phytochemicals, including flavonoids and iridoids, can moderately activate AHR to upregulate the expression of key epidermal differentiation proteins-filaggrin (FLG), loricrin (LOR), and involucrin (IVL) [19,20,21], which are crucial for epidermal differentiation and the construction of the physical barrier [22,23]. In our previous studies, GF ameliorated UVB-induced skin damage; however, its effects and underlying mechanisms in enhancing skin barrier function remain unclear [14].

In our study, the iridoid ingredients of GF were analyzed and identified via UPLC-MS/MS. Subsequently, the pro-migratory and cytoprotective activities of GF on HaCaT cells were validated using cell scratch wound healing, MTT, and ROS assays. To further investigate the effect of GF on the skin barrier, we employed proteomics analysis, molecular docking, and molecular dynamics (MD) simulations to elucidate potential targets and characterize GF’s binding interactions relevant to barrier function. The effects of GF on the expression of AHR, FLG, LOR, and IVL were confirmed via RT-qPCR and immunofluorescence assays. Finally, human efficacy assessments were conducted to test the efficacy of GF-containing gel formulation in enhancing skin barrier function by assessing skin moisture content, transepidermal water loss (TEWL), skin redness, and the lactic acid stinging test (LAST).

## 2. Results

### 2.1. Analysis of GF by UPLC-MS/MS

**Figure 1:**
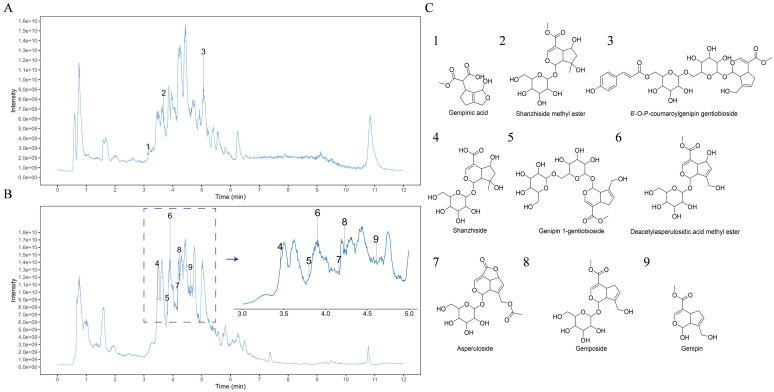
UPLC-MS/MS total ion chromatograms of the GF in positive (**A**) and negative (**B**) ion mode. (**C**) Structures of the identified constituents.

The positive and negative ion chromatograms of GF are presented in Figure 1A,B, respectively. Nine iridoids were identified in GF (Figure 1C, Figure S1, Table S1): (1) genipinic acid, (2) shanzhiside methyl ester, (3) 6’-O-P-coumaroylgenipin gentiobioside, (4) shanzhiside, (5) genipin-1-gentiobioside, (6) deacetylasperulosidic acid methyl ester, (7) asperuloside, (8) geniposide, and (9) genipin.

### 2.2. Effects of GF on Migration of HaCaT Cells

**Figure 2:**
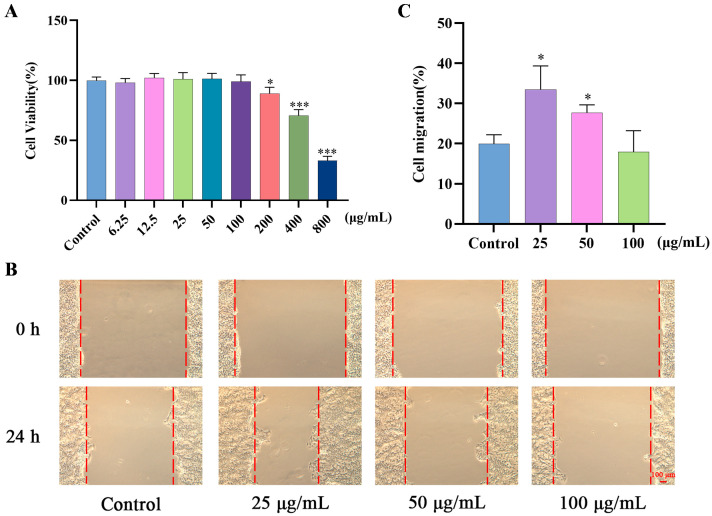
(**A**) HaCaT viability was measured by MTT assay; (**B**,**C**) images of the cell scratch assay were photographed under a microscope at 0 h and 24 h, and the scratch healing rates were measured by ImageJ 1.53q, scale bar = 100 μm; data (*n* = 3) are shown as mean ± SD. Compared with the control group: * *p* < 0.05 and *** *p* < 0.001.

The MTT assay was conducted to determine the cytotoxicity of GF. As shown in Figure 2A, GF exhibited no significant cytotoxicity at the concentrations of 6.25 to 100 μg/mL. Therefore, the maximum concentration of GF was limited to less than 100 μg/mL for subsequent experiments. Then, HaCaT cells were treated with 25, 50, or 100 μg/mL of GF for a scratch assay (Figure 2B,C). The result showed that GF significantly reduced the wound width at 25 μg/mL and 50 μg/mL after 24 h.

### 2.3. Protective Effects of GF on SLS-Induced HaCaT Cells

**Figure 3:**
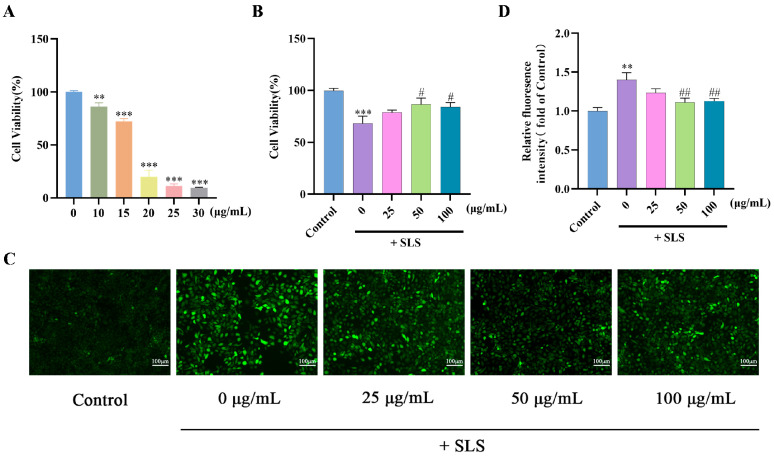
(**A**) HaCaT viability was measured by MTT assay; (**B**) GF increased the cell viability in SLS-treated HaCaT cells; (**C**,**D**) the ROS contents were stained with DCFH-DA, photographed by fluorescence microscopy and measured by ImageJ 1.53q, scale bar = 100 μm; data (*n* = 3) are shown as mean ± SD. Compared with the control group: ** *p* < 0.01 and *** *p* < 0.001. Compared with the SLS group: ^#^ *p* < 0.05 and ^##^ *p* < 0.01.

Sodium lauryl sulfate (SLS), an irritating surfactant, induces skin damage and sensitization [24,25]. As shown in Figure 3A, SLS at a concentration of 15 μg/mL was utilized to establish a damage model in HaCaT cells. The cell viability was reduced to 68.35% after SLS treatment, while GF co-treatment significantly inhibited the cytotoxicity induced by SLS (Figure 3B). Furthermore, GF also suppressed SLS-stimulated ROS generation (Figure 3C,D).

### 2.4. Proteomics Assay of GF on HaCaT Cells

**Figure 4:**
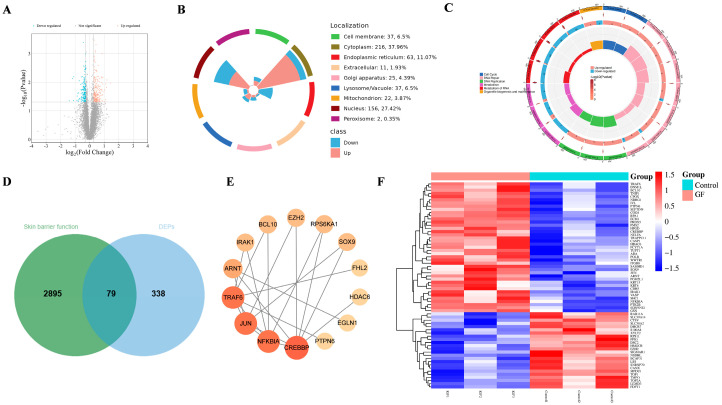
Analysis of proteomics. (**A**) Volcano plot comparing the effect of GF on HaCaT cells; (**B**) subcellular location of the DEPs; (**C**) Reactome pathway analysis of DEPs; (**D**) the intersection of targets between skin barrier function and DEPs; (**E**) PPI network analysis; (**F**) heatmap of skin barrier function-related DEPs.

To investigate the mechanisms by which GF regulates HaCaT cells, we performed proteomics analysis. Volcano plot analysis showed 417 differentially expressed proteins (DEPs) in response to GF treatment compared with the control group, with 239 upregulated and 178 downregulated (Figure 4A). Further analysis showed that GF primarily affected cell function by modulating cytoplasmic and nuclear proteins in HaCaT cells (Figure 4B). Furthermore, Reactome pathway analysis revealed that GF mainly affected cell function by modulating DNA repair, DNA synthesis, and metabolism within HaCaT cells (Figure 4C).

To further obtain skin barrier-related DEPs, targets associated with skin barrier function were collected from GeneCards, OMIM, and the CTD database and intersected with the DEPs. Venn diagram analysis showed 79 overlapping genes between the 2974 skin barrier function-related targets and 417 DEPs (Figure 4D). Based on the STRING database and Cytoscape 3.9.0, a protein–protein interaction (PPI) network displayed the key targets including CREBBP, NFKBIA, JUN, TRAF6, and ARNT (Figure 4E). The expression changes of these 79 potential targets were visualized using a heatmap (Figure 4F). Among these, targets associated with keratinocyte proliferation and differentiation, such as CREBBP, JUN, ARNT, SOX9, IVL, FHL2, KRT4, and KRT15, were upregulated by GF. ARNT is an essential ligand for the DNA-binding and transcriptional activity of the aryl hydrocarbon receptor (AHR), while IVL and SOX9 are known as AHR regulatory targets. Although AHR did not show significant upregulation in proteomics data, these results collectively suggest that AHR may be a potential target mediating GF’s effects on skin barrier function.

### 2.5. Molecular Docking

**Figure 5:**
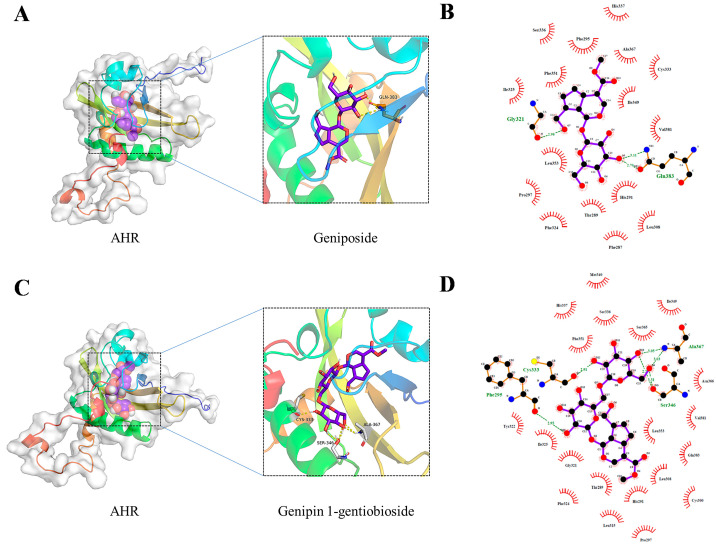
Molecular docking analysis of geniposide and genipin-1-gentiobioside with AHR. Visualization and 2D interaction diagram of molecular docking: (**A**,**B**) geniposide–AHR; (**C**,**D**), genipin-1-gentiobioside–AHR.

HPLC analysis identified geniposide and genipin-1-gentiobioside as the principal iridoids in GF [26]. In order to predict the potential interaction between GF and AHR, molecular docking was performed. The docked conformations and 2D interaction diagrams for the geniposide–AHR and genipin-1-gentiobioside–AHR complexes are shown in Figure 5A–D, respectively. Geniposide formed hydrogen bonds with Gly321 and Gln383 residues of AHR (Figure 5B), while genipin-1-gentiobioside established hydrogen bonds with four residues: Cys333, Ala367, Phe295, and Ser346 (Figure 5D). The mutual binding energies for these interactions are summarized in Table 1. Typically, a binding energy of less than −5 kcal/mol signifies an excellent binding affinity. The findings demonstrated a favorable binding activity between the ingredients and AHR, suggesting that GF may enhance skin barrier function via AHR activation.

### 2.6. MD Simulation

**Figure 6:**
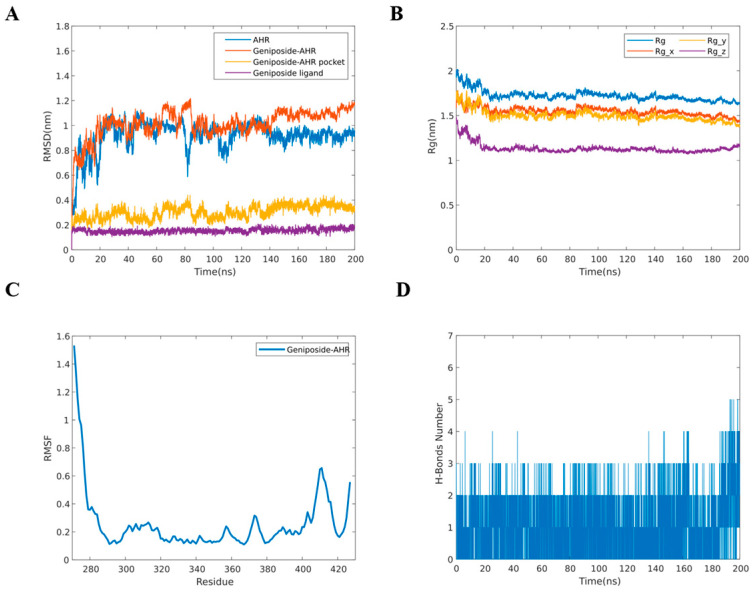
Results of MD simulations. (**A**) RMSD curves; (**B**) Rg curves; (**C**) RMSF curve; (**D**) number of hydrogen bonds.

Based on the content of ingredients and molecular docking scores, the geniposide–AHR complex was selected for MD simulations to assess binding stability. The positional deviations in the protein were analyzed using root-mean-square deviation (RMSD). The RMSD curve of the geniposide–AHR complex reached equilibrium after approximately 90 ns (Figure 6A). The analysis of the RMSD values for the geniposide and AHR pocket indicated that the active pocket of the small molecule and protein was in a stable state. These findings suggest that the binding of geniposide did not induce significant conformational changes in AHR, resulting in a stable complex. The radius of gyration (Rg) indicates the tightness of binding of the system. The data showed that the geniposide–AHR complex exhibited stable rotation radii and fluctuated around 1.7 nm (Figure 6B). Root-mean-square fluctuation (RMSF) is used to reflect the fluctuation of amino acid residues during the simulation process. Figure 6C demonstrated that the RMSF values of amino acid residues in the head and tail regions were relatively high, while the overall fluctuations were not large (0.1 nm to 0.4 nm), indicating the stability of the formed complex. The number of hydrogen bonds reflected the binding stability of the complex. As shown in Figure 6D, the maximum number of hydrogen bonds in the geniposide–AHR complex was 5, with the majority of hydrogen bonds concentrated between 1 and 2 [27].

These MD simulations demonstrated that geniposide forms a stable complex with AHR. This provides robust computational evidence that geniposide, the primary active component of GF, activates the AHR pathway to enhance skin barrier function.

### 2.7. Effects of GF on AHR/FLG/LOR/IVL Gene Expression in HaCaT Cells

**Figure 7:**
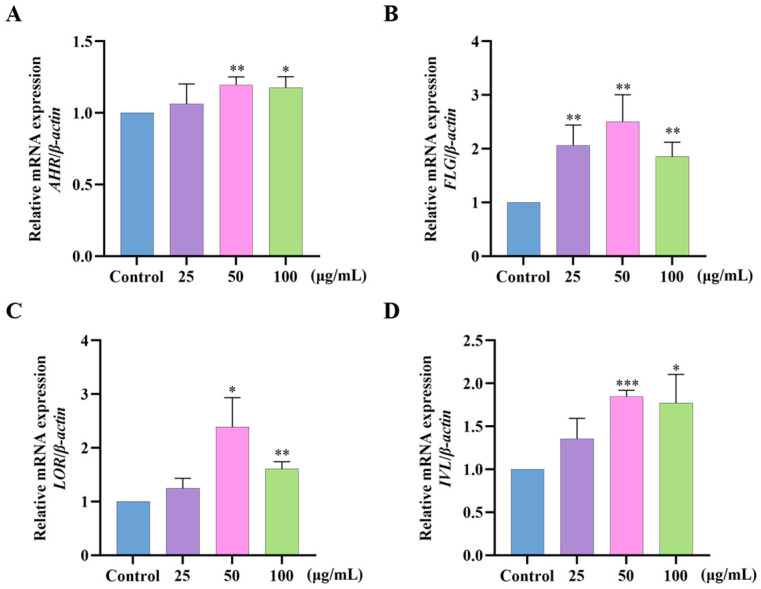
Effect of GF on mRNA expression of *AHR*, *FLG*, *LOR,* and *IVL*. The mRNA expression levels of (**A**) *AHR*, (**B**) *FLG*, (**C**) *LOR,* and (**D**) *IVL* were measured by RT-qPCR. Data (*n* = 3) are shown as mean ± SD. Compared with the control group: * *p* < 0.05, ** *p* < 0.01, and *** *p* < 0.001.

To test whether GF could improve the skin barrier by altering AHR, the mRNA expression levels of *AHR*, *FLG*, *LOR*, and *IVL* were determined in HaCaT cells (Figure 7). GF effectively upregulated the mRNA levels of *AHR*, *FLG*, *LOR,* and *IVL* compared with the control groups. Meanwhile, the expression of *AHR*, *FLG*, *LOR*, and *IVL* showed a consistent trend, suggesting GF may enhance skin barrier function through a moderately activated AHR signaling pathway.

### 2.8. Effects of GF on FLG/LOR/IVL Expression in 3D Epidermal Skin Models

**Figure 8:**
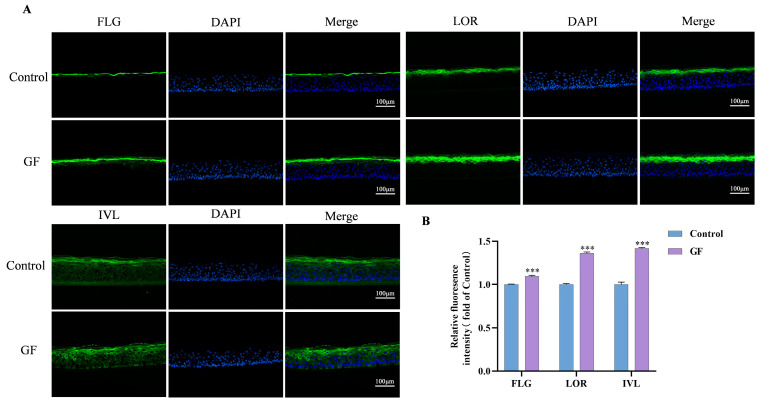
Effect of GF on protein expression of FLG, LOR, and IVL in epidermal skin models. The protein expression levels of FLG, LOR, and IVL were stained by immunofluorescence (**A**) and measured by ImageJ 1.53q, (**B**), scale bar = 100 μm; data (*n* = 3) are shown as mean ± SD. Compared with the control group: *** *p* < 0.001.

FLG, LOR, and IVL are specific proteins expressed mainly in the granular and stratum corneum layers of the skin [22]. In order to accurately validate the impact of GF on FLG, LOR, and IVL expression, immunofluorescence staining was performed. FLG, LOR, and IVL expression in the epidermis was compared after the application of 50 μg/mL GF (Figure 8). The FLG, LOR, and IVL expression levels were effectively increased by GF compared to the control.

### 2.9. Participants’ Characteristics

Thirty-one healthy Chinese women and three healthy Chinese men participated in the 4-week randomized, double-blind, prospective clinical study, in which all subjects had a positive LAST (total score ≥ 3). The average age of the participants was 49.5 ± 11.4 years.

### 2.10. Effects of GF on Skin Hydration and TEWL

**Figure 9:**
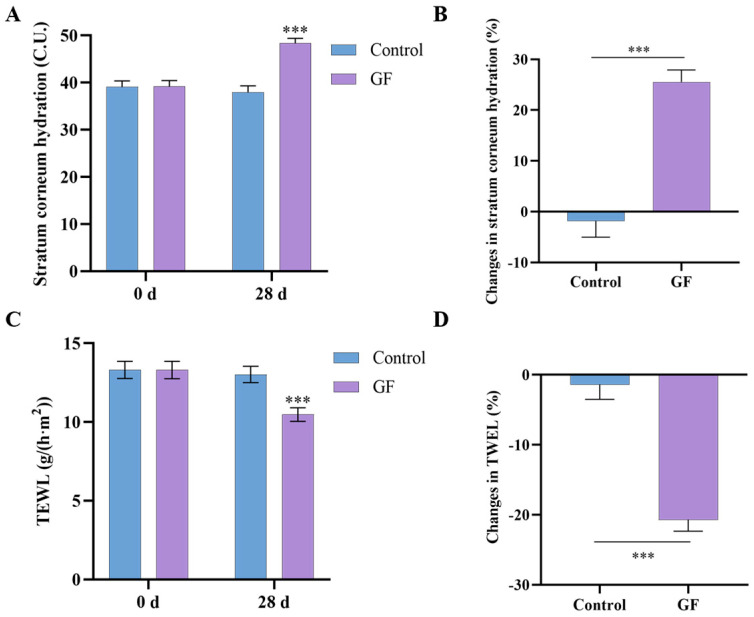
Human efficacy assessments over 28-day treatment with GF vs. placebo. (**A**,**B**) Stratum corneum hydration; (**C**,**D**) TEWL; data (*n* = 17) are shown as mean ± SEM. Compared with the placebo group or 0 d: *** *p* < 0.001.

Skin hydration and TEWL were measured using the Corneometer CM 825 and Tewameter TM 300, respectively. Following a 28-day period, the GF group showed a significant increase in stratum corneum hydration from 39.20 ± 7.18 to 48.33 ± 6.09 (25.54%, *p* < 0.001) (Figure 9A,B), and a significant decrease in TEWL from 13.31 ± 3.19 to 10.48 ± 2.50 (20.73%, *p* < 0.001) (Figure 9C,D). In contrast, the placebo group exhibited no significant changes in either skin hydration or TEWL. As skin hydration and TEWL are the most widely used parameters for assessing skin barrier function, the comparison between groups demonstrated that the GF group showed a significantly greater improvement in skin barrier function after 28 days [28].

### 2.11. Effects of GF on Skin Redness and Tolerability

**Figure 10:**
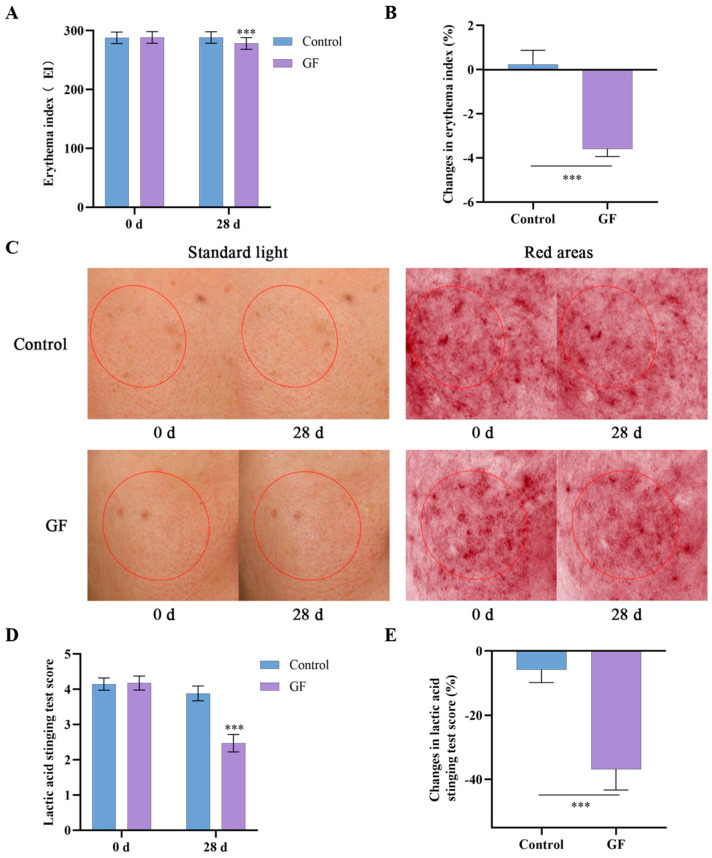
Human efficacy assessments over 28-day treatment with GF vs. placebo. (**A**,**B**) Erythema index; (**C**) VISIA; (**D**,**E**) lactic acid sting tests; data (*n* = 17) are shown as mean ± SEM. Compared with the placebo group or 0 d: *** *p* < 0.001.

To investigate the effects of GF on skin sensitization and skin tolerance, the erythema index (E.I.) was measured using the Mexameter MX 18, and a LAST was performed. The application of the gel containing GF reduced skin redness after 28 days, with a slightly lower E.I. value on the treated side compared with baseline (Figure 10A–C). In contrast, the placebo group showed no significant improvement in E.I. value. The LAST exhibited a reduction of 36.81% in the test area after 28 days, while the control area demonstrated a 5.78% decline (Figure 10D,E). Furthermore, no adverse reactions were observed in any of the 34 subjects during the trial period. These findings indicate that GF has the potential to strengthen the skin barrier, contributing to reduced skin sensitivity and improved tolerance.

## 3. Discussion

Natural plant extracts are increasingly utilized in dermatological cosmetics. GF, a traditional Chinese medicine with skin-improving properties, has garnered our attention. GF exhibits significant effects in detoxification, reducing swelling, and alleviating pain. Clinically, it has long been used for the repair of damaged skin tissue. Iridoids, a special class of monoterpenoids found in GF, serve as major bioactive components with numerous pharmacological activities. In this study, nine iridoids in GF were identified via UPLC-MS/MS. Compared to our previous experiments, three additional iridoid compounds were identified in GF: genipinic acid, 6′-O-P-coumaroylgenipin gentiobioside, and deacetylasperulosidic acid methyl ester [14].

Keratinocytes, the predominant cell type in the epidermis, play a pivotal role by migrating, proliferating, and differentiating to form the skin barrier [29,30]. Studies have revealed that the viability and migration of keratinocytes directly influence the re-epithelialization process [31]. Consequently, enhancing keratinocyte functional activity associated with wound healing, such as migration and proliferation, represents a strategy for repairing skin damage and reinforcing barrier function [32]. Consistent with this approach, our results confirmed that GF significantly promoted HaCaT cell migration, supporting its barrier-reinforcing potential. Additionally, when exposed to external stressors such as ultraviolet (UV) radiation, SLS, and environmental pollutants, keratinocytes initiate a defense response by inflammatory cytokine production and nociceptive signaling modulation [33,34,35]. GF treatment effectively suppressed the reduction in cell viability induced by SLS, and inhibited the fluorescence intensity of intracellular ROS to reduce cell barrier damage. In conclusion, the findings indicated that GF possesses the capacity to repair and strengthen the skin barrier.

Previous studies have demonstrated that GF improved UVB-induced barrier damage and promoted ZO-1 expression [14,26,36]. However, the underlying mechanism by which GF enhances barrier function remains unclear. To further investigate the potential effects of GF on skin barrier function, we conducted proteomics combined with network pharmacology, molecular docking, and MD. In our study, the expression of 417 proteins was significantly altered after GF treatment, and 79 targets may be involved in regulating skin barrier function. PPI network analysis showed that the immunity- and inflammation-related targets, such as NFKBIA, JUN, and TRAF6, were essential targets for GF-mediated barrier-improving effects. Notably, ARNT caught our attention. ARNT is a pivotal protein that forms heterodimers with AHR and initiates the expression of multiple biotransformation enzyme genes [37]. Heatmap analysis also revealed that GF significantly upregulated ARNT expression. Although AHR showed a non-significant upward trend, the collective findings suggest AHR may be a potential target for GF-mediated barrier enhancement. To validate this hypothesis, molecular docking and MD simulations were conducted. In our research, the main iridoids of GF exhibited favorable binding activities with AHR, and the geniposide–AHR complex showed stable binding in MD simulations.

The process of epithelial differentiation is characterized by the upregulation of specific proteins’ expression, including FLG, LOR, and IVL, that interact to form a cornified envelope and participate in the construction of the skin barrier [38]. Proteomics studies further revealed proteins associated with keratinocyte proliferation and differentiation, such as SOX9, IVL, FHL2, KRT4, and KRT15, whose expression levels were significantly elevated. Interestingly, many antioxidant phytochemicals can exert their antioxidant effects via the activation of AHR and NRF2, while regulating keratinocyte differentiation and barrier function through the moderate activation of AHR [39,40,41]. GF has shown effective antioxidant capacity, which has the potential to modulate AHR to improve skin barrier function. Subsequently, the effects of GF on barrier-related targets were validated by RT-qPCR and immunofluorescence. RT-qPCR results confirmed that GF upregulated the expression of AHR, FLG, LOR, and IVL, with the expression trends of FLG, LOR, and IVL consistent with that of AHR. In 3D epidermal models, immunofluorescence localized FLG/LOR predominantly in the granular layer and IVL in spinous/granular layers, while GF treatment significantly increased FLG/LOR/IVL expression.

It is commonly accepted that alterations in stratum corneum hydration and TEWL reflect changes in skin barrier function [42,43]. Higher skin moisture content and lower TEWL values are indicative of better stratum corneum barrier function. In our human efficacy assessments, the active gel containing GF showed excellent moisturizing and barrier-improving properties. The average stratum corneum hydration of participants increased by 25.54% and the average TEWL of participants decreased by 20.73% after applying the active gel over a 28-day period. Additionally, the levels of erythema index and LAST score are frequently employed to assess vascular reactivity or inflammatory responsiveness, which can reflect barrier function to some extent [44,45]. The active gel significantly reduced both erythema index and LAST scores, with excellent tolerability and no adverse events reported. The results of this study lay the foundation for further development of GF as an active cosmetic ingredient for skin care products.

## 4. Materials and Methods

### 4.1. Preparation and Analysis of GF

Gardeniae Fructus (GF) is the fruit of *Gardenia jasminoides* Ellis purchased from Fujian Zhiyu Agricultural Development Co., Ltd. (Ningde, China) and authenticated by Prof. Wu (Kunming Institute of Botany, Chinese Academy of Sciences). The plant material was weighed, powdered, and extracted twice with 20 volumes of water at 80 °C for 2 h. Then, the filtrates were combined, decolorized, purified, and desorbed to enrich iridoids. The solution was concentrated and freeze-dried to obtain the GF extract with a yield of 6.98%, and the contents of genipin-1-gentiobioside and geniposide were determined to be 148.0 mg/g and 487.1 mg/g, respectively, in a previous experiment [25].

UPLC-MS/MS analyses were performed using a UHPLC system (Vanquish, Thermo Fisher Scientific, Waltham, MA, USA) equipped with a Phenomenex Kinetex C18 column (2.1 × 100 mm, 2.6 μm) coupled to an Orbitrap Exploris 120 mass spectrometer (Thermo Fisher Scientific). MS/MS spectra were acquired in information-dependent acquisition (IDA) mode using Xcalibur 4.3 software (Thermo Fisher Scientific). Compounds were identified by spectral matching against a proprietary database (BIOTREE Biomedical Technology Co., Ltd., Shanghai, China) using fragmentation pattern matching algorithms (Table S1).

### 4.2. Cell Culture

Human keratinocytes (HaCaT) were obtained from Zhejiang Meisen Cell Technology Co., Ltd. HaCaT cells were cultured in DMEM (Gibco, Carlsbad, CA, USA) supplemented with 10% FBS and 1% penicillin/streptomycin at 37 °C in a 5% CO_2_ incubator.

### 4.3. Cell Viability Assay

HaCaT cells were treated with various concentrations of GF for 24 h. Then, cells were incubated with MTT (0.5 mg/mL, Beyotime Biotechnology, Shanghai, China). After 4 h, the formazan was dissolved in dimethyl sulfoxide (DMSO, Sinopharm, Shanghai, China) and the absorbance was measured at 570 nm using Infinite E Plex (Tecan, Männedorf, Switzerland).

### 4.4. Cell Scratch Assay

HaCaT cells were seeded into six-well plates and cultured to 100% confluence. Subsequently, the cells were washed with PBS and scratched using a 200 µL sterile pipette tip. After scratching, 25, 50, or 100 μg/mL GF was added to the wells and incubated for 24 h. Images were acquired at 0 h and 24 h via a microscope (Motic, Xiamen, China), and the scratch gap width was measured by ImageJ.

### 4.5. Detection of Reactive Oxygen Species (ROS) Production

The levels of intracellular ROS were measured using DCFH-DA (Beyotime Biotechnology, Shanghai, China). HaCaT cells were pretreated with GF and/or SLS for 24 h and incubated with 10 µM DCFH-DA for 25 min at 37 °C. The cells were observed under a fluorescence microscope (Motic, Xiamen, China).

### 4.6. Proteomics Assay

HaCaT cells were treated with GF at a concentration of 50 µg/mL for 24 h prior to protein extraction. Proteomics sample preparation and assay are described in the Supplementary Materials (Methods S1). Differentially expressed proteins (DEPs) were identified using a fold change greater than 1.2 and a *p*-value less than 0.05.

“Skin barrier function” was used as the keyword to obtain, merge, and deduplicate targets from GeneCards (https://www.genecards.org/, accessed on 1 July 2024), Online Mendelian Inheritance in Man (OMIM, https://omim.org/, accessed on 1 July 2024), and Comparative Toxicogenomics Database (CTD, https://ctdbase.org/, accessed on 1 July 2024). Then, a Venn diagram of skin barrier function-related targets and DEPs was drawn on the bioinformatics cloud platform. The PPI network was analyzed based on the STRING database (https://www.string-db.org/, accessed on 1 July 2024), limiting the species to “Homo sapiens” and using confidence data > 0.7, and visualized by Cytoscape 3.9.0. A heatmap of DEPs associated with skin barrier function was plotted by the bioinformatics cloud platform.

### 4.7. Molecular Docking

The protein structure was downloaded from the PDB database with the code 7ZUB, and ingredient structures were obtained from the PubChem database. AutoDock Vina 1.25 was employed for the molecular docking process. For the geniposide–AHR and genipin-1-gentiobioside–AHR complexes, the docking center coordinates were set as follows: center_x = 160.767, center_y = 163.935, center_z = 159.496. The box size was set to a cube with dimensions size_x = 16.33, size_y = 16.33, size_z = 16.33. The results were plotted and displayed using PyMol 2.3.0.

### 4.8. MD Simulation

MD simulations were performed using GROMACS software (version 2024.3). The AMBER force field was used to generate the ligand topology file by the ACPYPE script, while the protein topology file was created using the AMBER99SB force field. In the MD simulations, the system was neutralized with Na^+^ ions and Cl^−^ ions, and the complex was minimized through the steepest descent algorithm and conjugate gradient method. Then, MD dynamics simulations were performed under periodic boundary conditions at 310 K and 1.0 bar for 200 ns. Data were analyzed using GROMACS [46].

### 4.9. RT-qPCR

RNA-easy Isolation Reagent was used to extract total RNA from HaCaT cells, and RNA was reverse transcribed into cDNA by HiScript III RT SuperMix for qPCR (Vazyme, Nanjing, China). CFX Duet (Bio-Rad, Hercules, CA, USA) and ChamQ SYBR qPCR Master Mix (Vazyme, Nanjing, China) were then applied to determine the mRNA expression of *AHR*, *FLG*, *LOR*, and *IVL*. Primers used in this study are provided in Table 2.

### 4.10. Immunofluorescence Staining

Three-dimensional epidermal skin models (Regenovo, Hangzhou, China) were incubated with 50 μg/mL GF for 24 h at 37 °C. Then, the skin models were collected for frozen sectioning. After rewarming, sections were blocked and incubated with primary antibodies, including FLG, LOR, and IVL (Abcam, Cambridge, Cambridgeshire, UK), overnight. After washing with PBS/T, the sections were incubated with Alexa Fluor 488-conjugated secondary antibodies (Abcam, Cambridge, Cambridgeshire, UK) and DAPI (Beyotime Biotechnology, Shanghai, China). The sections were observed under a fluorescent inverted microscope and analyzed via ImageJ software.

### 4.11. Volunteer Tests

We conducted a human efficacy assessment on 34 volunteers aged 20–60 years. They were randomly divided into two groups: Group 1 received the placebo gel, while Group 2 received the GF gel (Table 3). Before the volunteer tests, the volunteers were informed about the purpose and procedure of the tests, agreed to participate, and signed a written informed consent form. The cosmetic formulations tested included the placebo gel and the GF gel to be applied twice a day, in the morning and at night. During the entire test period, sun exposure, outdoor sports, and travelling were to be avoided. In addition, the application of cosmetics or medicines with similar efficacy was prohibited, and no changes to the subject’s daily care routine were allowed.

Skin conditions were measured before the application of the products on the day this study started (0 d) and after 28 days (28 d) of use. Corneometer CM 825, Tewameter TM 300, and Mexameter MX 18 (Courage + Khazaka, Cologne, Germany) were used to assess skin parameters. The LAST was performed by trained research professionals, who asked the subjects about their self-perceived symptoms, which were scored and recorded on a 4-point scale.

### 4.12. Statistical Analysis

Statistical analysis was performed using GraphPad Prism 9 software. Differential analyses were calculated by Student’s *t*-test, and *p* < 0.05 was considered statistically significant.

## 5. Conclusions

In conclusion, GF significantly promoted epidermal cell migration and enhanced cellular resistance to SLS-induced oxidative damage. Integrated proteomics, molecular docking, MD simulations, and in vitro verification indicated that GF treatment improved skin barrier function by modulating AHR-mediated upregulation of FLG/LOR/IVL. Clinically, a 28-day randomized trial demonstrated that GF gel effectively increased average skin hydration by 25.54%, reduced average TEWL by 20.73%, and alleviated skin sensitivity. These multi-level findings establish GF as a potential functional ingredient that enhances the skin barrier via FLG/LOR/IVL induction, supporting its translation to skin beauty and healthcare applications. Meanwhile, the skin barrier-enhancing components and the mechanisms of GF still need further investigation.

## Figures and Tables

**Table 1:** Molecular docking scores of the ingredients and AHR.

Ingredient	Docking Score (Kcal/Mol)
geniposide	−7.6
genipin-1-gentiobioside	−5.1

**Table 2:** Primers for RT-qPCR.

Gene	Forward Sequence (5′-3′)	Reverse Sequence (5′-3′)
*β-actin*	TGGCACCCAGCACAATGAA	CTAAGTCATAGTCCGCCTAGAAGCA
*AHR*	CAAATCCTTCCAAGCGGCATA	CGCTGAGCCTAAGAACTGAAAG
*FLG*	TTCGGCAAATCCTGAAGAATCC	ACTGTGCTTTCTGTGCTTGTG
*LOR*	GGAGATCAGTGCTCCTCACA	AGCAGAACTAGATGCAGCCG
*IVL*	GGGTATTGACTGGAGGAGGAACA	AGCCTTACTGTGAGTCTGGTTGA

**Table 3:** Cosmetic formulations.

Ingredient	Placebo Gel	GF Gel
WATER	94.20%	92.60%
PENTYLENE GLYCOL	3%	3%
GF	/	2%
GLYCERIN	2%	2%
ACRYLATES/C10-30 ALKYL ACRYLATE CROSSPOLYMER	0.35%	0.35%
ARGININE	0.25%	0.25%
HYDROXYACETOPHENONE	0.20%	0.20%

## Data Availability

The data that support the findings of this study are available from the corresponding author upon reasonable request.

## Supplementary Materials

The following supporting information can be downloaded at https://www.mdpi.com/article/10.3390/molecules30183764/s1: Table S1: Nine iridoids identified in GF. Figure S1: Nine iridoids identified in GF.

## Abbreviations

The following abbreviations are used in this manuscript:

GF	Gardeniae Fructus
AHR	Aryl hydrocarbon receptor
FLG	Filaggrin
LOR	Loricrin
IVL	Involucrin
HPLC	High-performance liquid chromatography
TEWL	Transepidermal water loss
E.I.	Erythema index
LAST	Lactic acid stinging test
HaCaT	Human keratinocytes
ROS	Reactive oxygen species
SLS	Sodium lauryl sulfate
DEPs	Differentially expressed proteins
MD	Molecular dynamics
RMSD	Root-mean-square deviation
RMSF	Root-mean-square fluctuation
Rg	Radius of gyration
