# Supplementary material for: Gardeniae Fructus Enhances Skin Barrier Function via AHR-Mediated FLG/LOR/IVL Expression
Source: Molecules. 2025 Sep 16;30(18):3764. doi: 10.3390/molecules30183764 (PMC12472589; doi:10.3390/molecules30183764)
Supplement: Supplementary file 1 [file molecules-30-03764-s001.zip › Methods S1.pdf]

Supplementary Methods

1.1 Cell lysis and proteome sample preparation

For each tube with cell precipitate, 30 μL of lysis buffer containing 6 M urea and 2 M thiourea was introduced. The contents were then transferred to a pressure cycling technology (PCT) tube. Subsequently, 5 μL of tris(2-carboxyethyl) phosphine (TCEP) at a concentration of 200 mM and 2.5 μL of iodoacetamide (IAA) at 800 mM, both dissolved in 100 mM Triethylammonium bicarbonate buffer (TEAB). The reduction and alkylation were conducted under 45000 psi, with 30 s high pressure and 10 s ambient pressure per cycle, 30 °C for 90 cycles. Then 10 μL Trypsin (0.5 μg/μL) and 2.5 μL rLys-C (0.5 μg/μL) were added for protein digestion under 20000 psi, with 50 s high pressure and 10 s ambient pressure per cycle, 30 °C for 120 cycles. Tryptic peptides were transferred into 1.5 mL tubes and digestion was then terminated by 15 μL 10% trifluoroacetic acid (TFA).

1.2 Desalting

Confirm that the pH of the samples was between 2 and 3. The SOLAμTM SPE Plate (Thermo Fisher ScientificTM, San Jose, USA) was applied for desalting according to the user guides provided by the producer.

1.3 LC-MS/MS

Liquid chromatography-mass spectrometry (LC-MS) analysis was performed using a Vanquish Neo UHPLC system coupled to an Orbitrap Astral mass spectrometer (Thermo Scientific, San Jose, USA) for data-independent acquisition (DIA) analysis. The mobile phase A consisted of 98% water with 0.1% formic acid and 2% Acetonitrile. While, mobile phase B comprised 20% water with 80% acetonitrile and 0.1% formic acid. All reagents were of MS grade.

For the DIA acquisition, the peptide concentration was set to 0.2 μg/μL, with an injection volume of 1 μL. The amount of sample loaded for each DIA acquisition was 200 ng.

During sample acquisition, peptides were loaded onto a pre-column (5 μm, 5

mm*300 μm i.d.) at a pressure of 800 bar, then eluted onto an analytical column (1.9 μm, 120 Å, 150 mm*75 μm i.d.) at a flow rate of 2 μL/min. An 5.7 minutes effective LC gradient (12% to 45% mobile phase B) was used for analysis.

The mass spectrometry scan parameters were configured as follows: FAIMS voltage was set to -42 V. The primary scan was conducted over a range of 380 to 980 m/z, with a resolution of 240,000. The normalized AGC target was set to 500%, and the maximum injection time (max IT) was 5 ms. For secondary scans, the m/z range was adjusted to 150-2000, maintaining the normalized AGC target at 500%. The collision energy was calibrated to 25%, with a maximum injection time of 3 ms. The precursor ion mass range for these scans was specified as 380-980 m/z, with an isolation window of 2 m/z and no overlap between windows. A total of 300 windows were utilized for the analysis.

1.4 Mass spectrometry data analysis

The mass spectrometry data were processed using the DIA-NN software (version 1.8.1) for database searching, with the match-between-run (MBR) feature enabled, utilizing the uniprot fasta files(2023-07-25-reviewed-contam-UP000005640_human_pd.fasta). Carbamidomethylation of cysteine was set as static modifications, while oxidation of methionine was set as variable modifications. This comprehensive analysis provided both qualitative and quantitative data, applying a stringent threshold for the false discovery rate (FDR) of less than 0.01 as the filtering criterion.

1.5 Statistical and bioinformatics analysis

All the statistical and bioinformatics was performed in R (v.4.0). Missing value was imputed with 0. Principal Components Analysis (PCA) was conducted to visualize the separation of each groups using the stats package in R.

R package clusterProfiler was used to perform the Gene ontology (GO) and Kyoto Encyclopedia of Genes and Genomes (KEGG) enrichment analysis. GSEA was performed by the R/Bioconductor package clusterProfiler.
