# Supplementary figures and images for: Gardeniae Fructus Enhances Skin Barrier Function via AHR-Mediated FLG/LOR/IVL Expression
Source: Molecules. 2025 Sep 16;30(18):3764. doi: 10.3390/molecules30183764 (PMC12472589; doi:10.3390/molecules30183764)

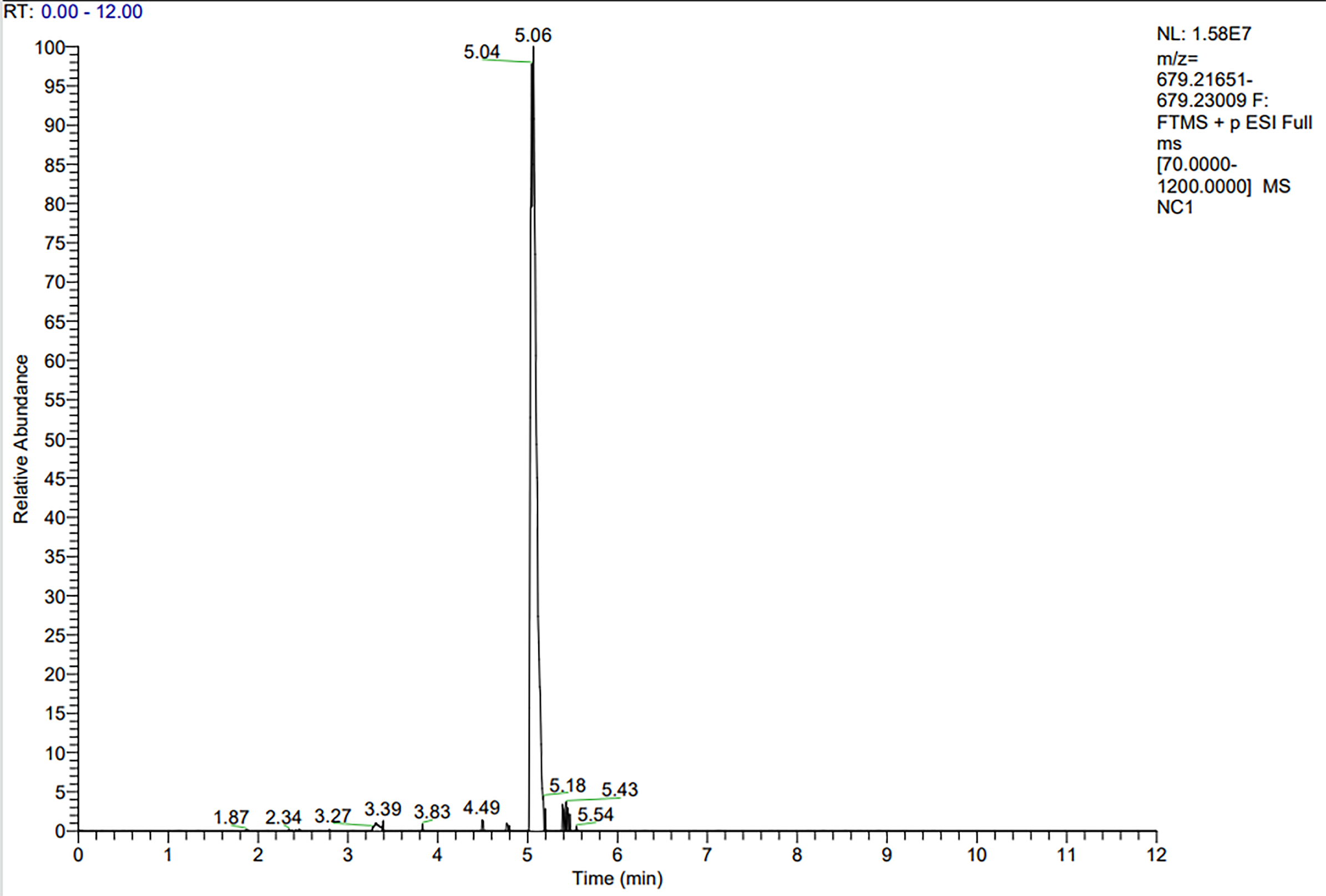

Supplement: Supplementary file 1 [file molecules-30-03764-s001.zip › Supplementary Figure S1/6'-O-P-coumaroylgenipin gentiobioside.png]

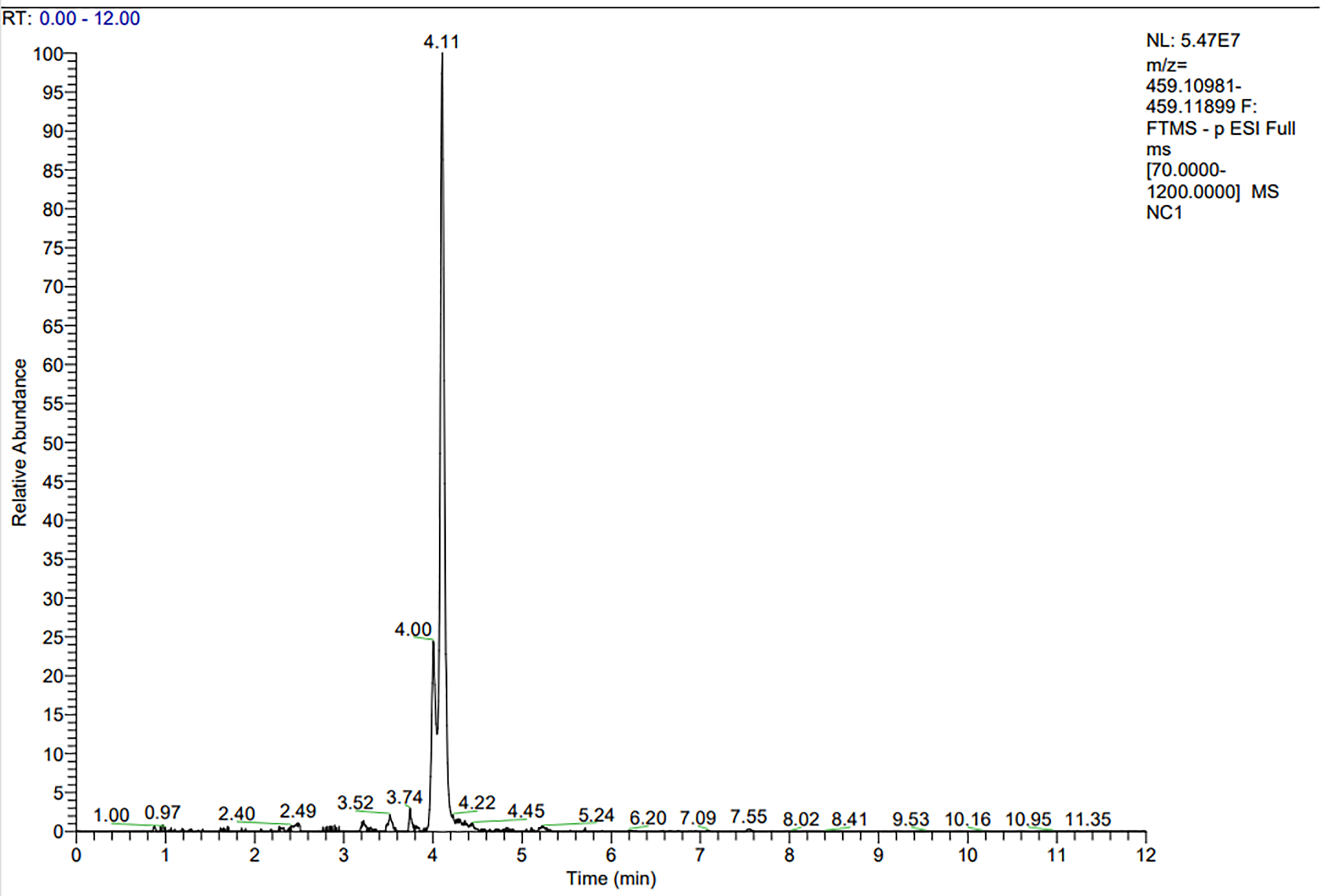

Supplement: Supplementary file 1 [file molecules-30-03764-s001.zip › Supplementary Figure S1/Asperuloside.png]

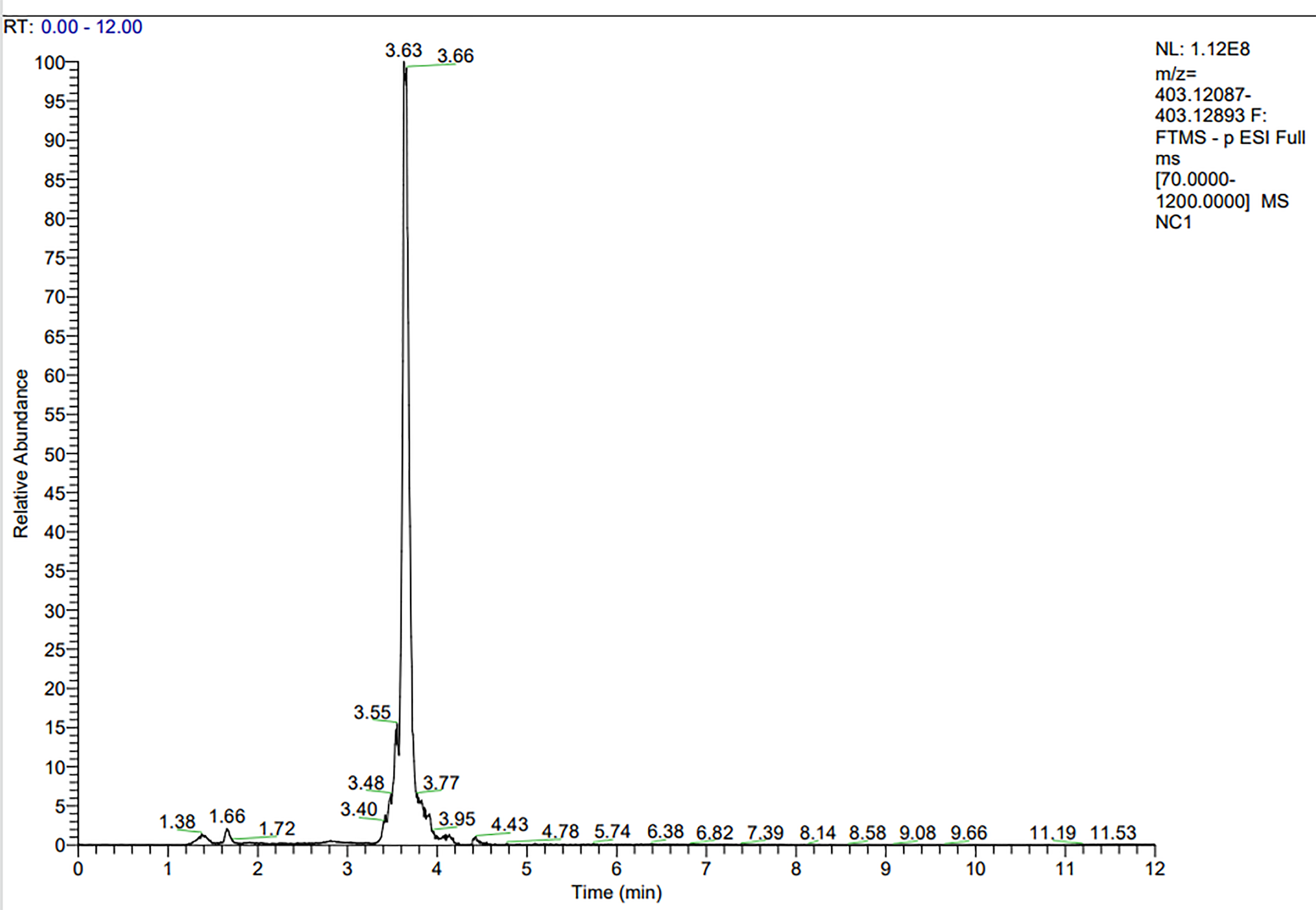

Supplement: Supplementary file 1 [file molecules-30-03764-s001.zip › Supplementary Figure S1/Deacetylasperulosidic acid methyl ester.png]

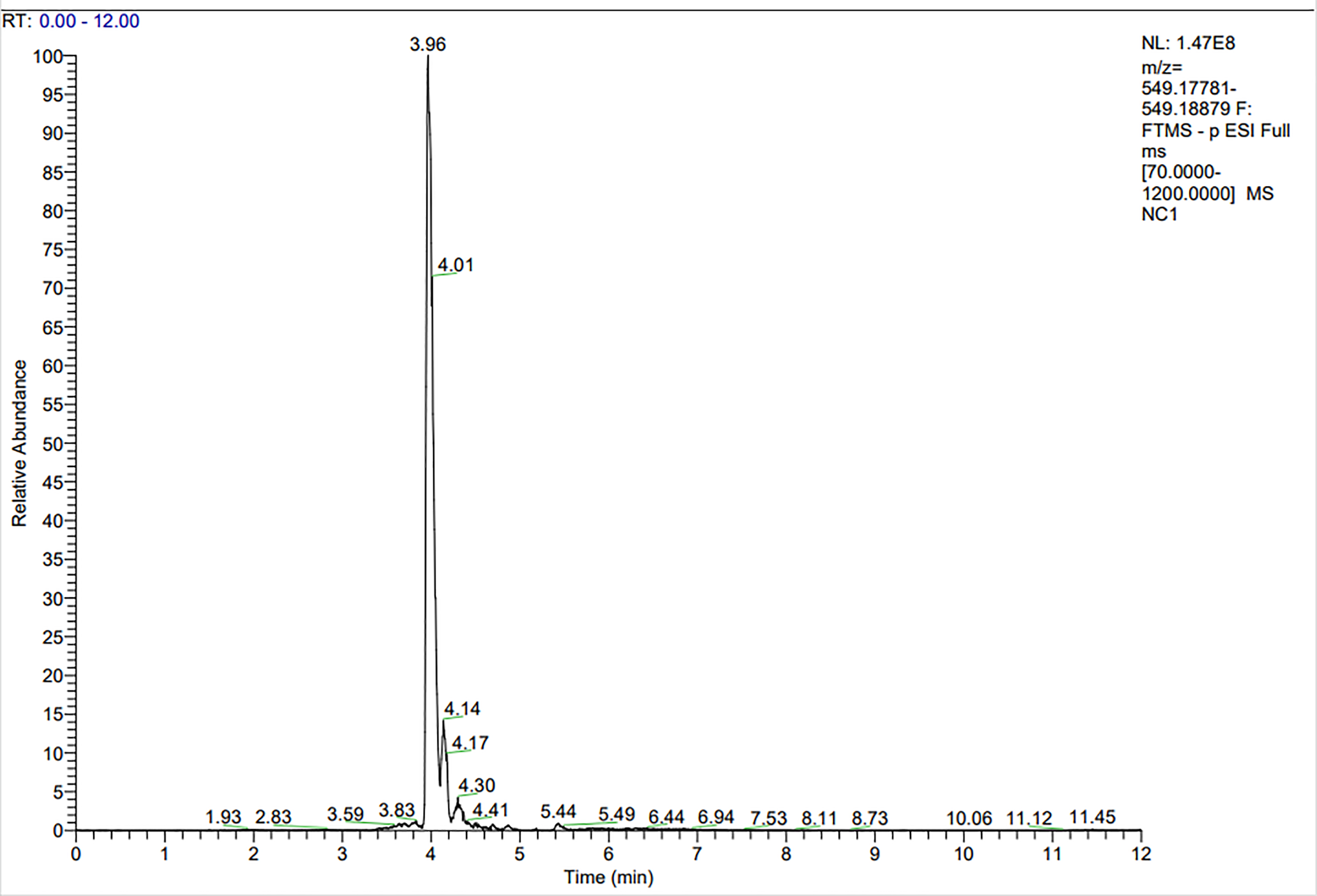

Supplement: Supplementary file 1 [file molecules-30-03764-s001.zip › Supplementary Figure S1/Genipin 1-gentiobioside.png]

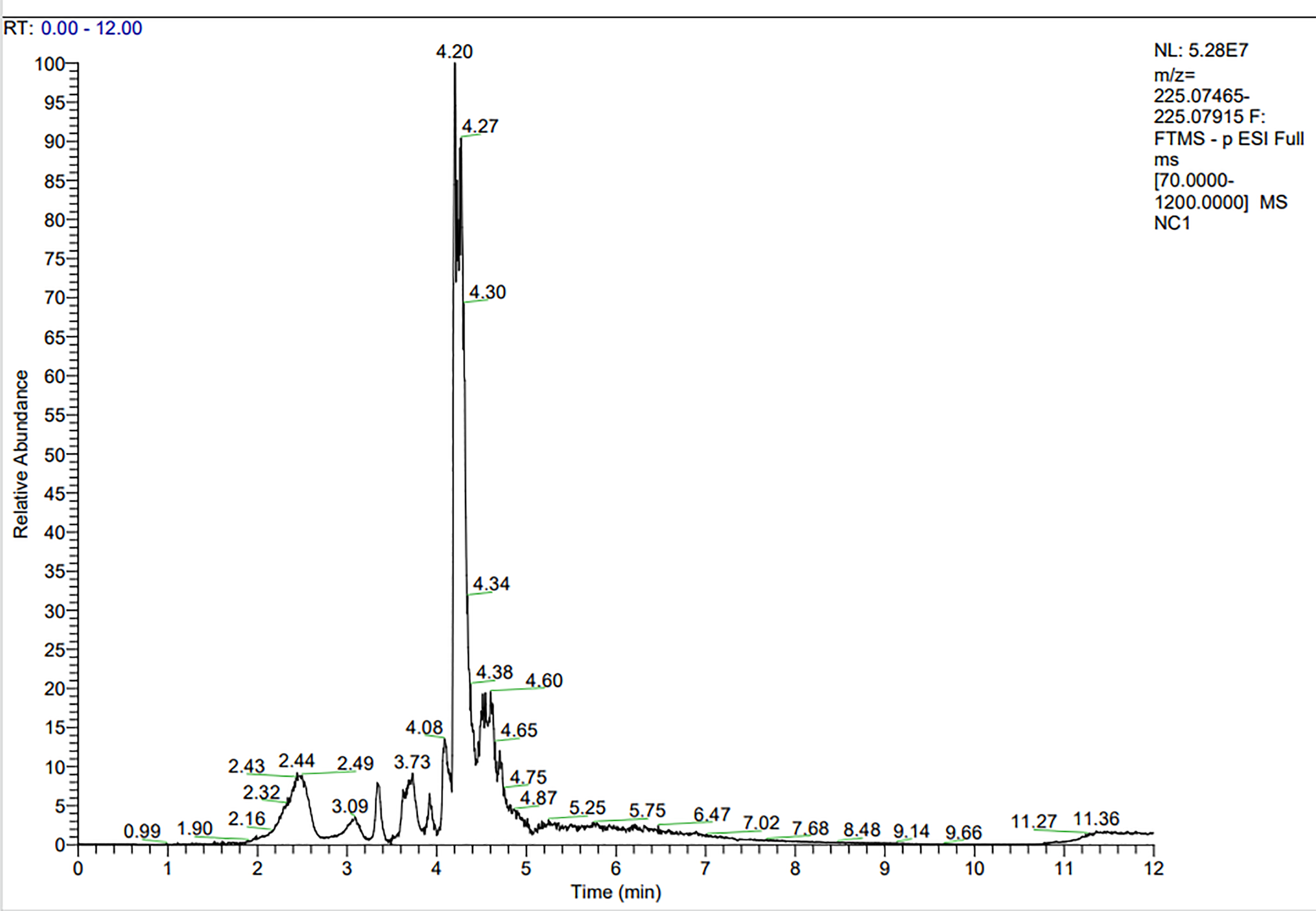

Supplement: Supplementary file 1 [file molecules-30-03764-s001.zip › Supplementary Figure S1/Genipin.png]

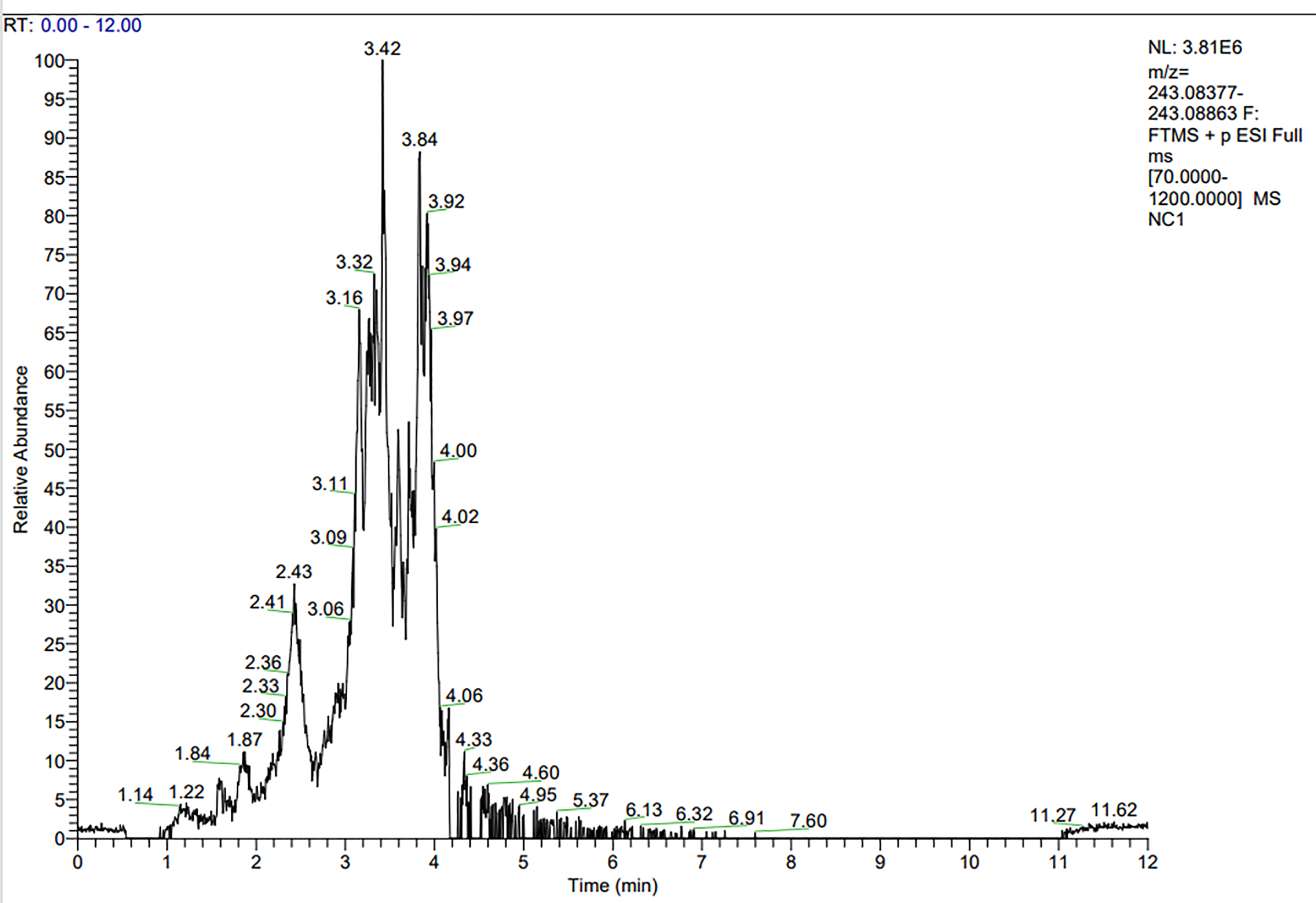

Supplement: Supplementary file 1 [file molecules-30-03764-s001.zip › Supplementary Figure S1/Genipinic acid.png]

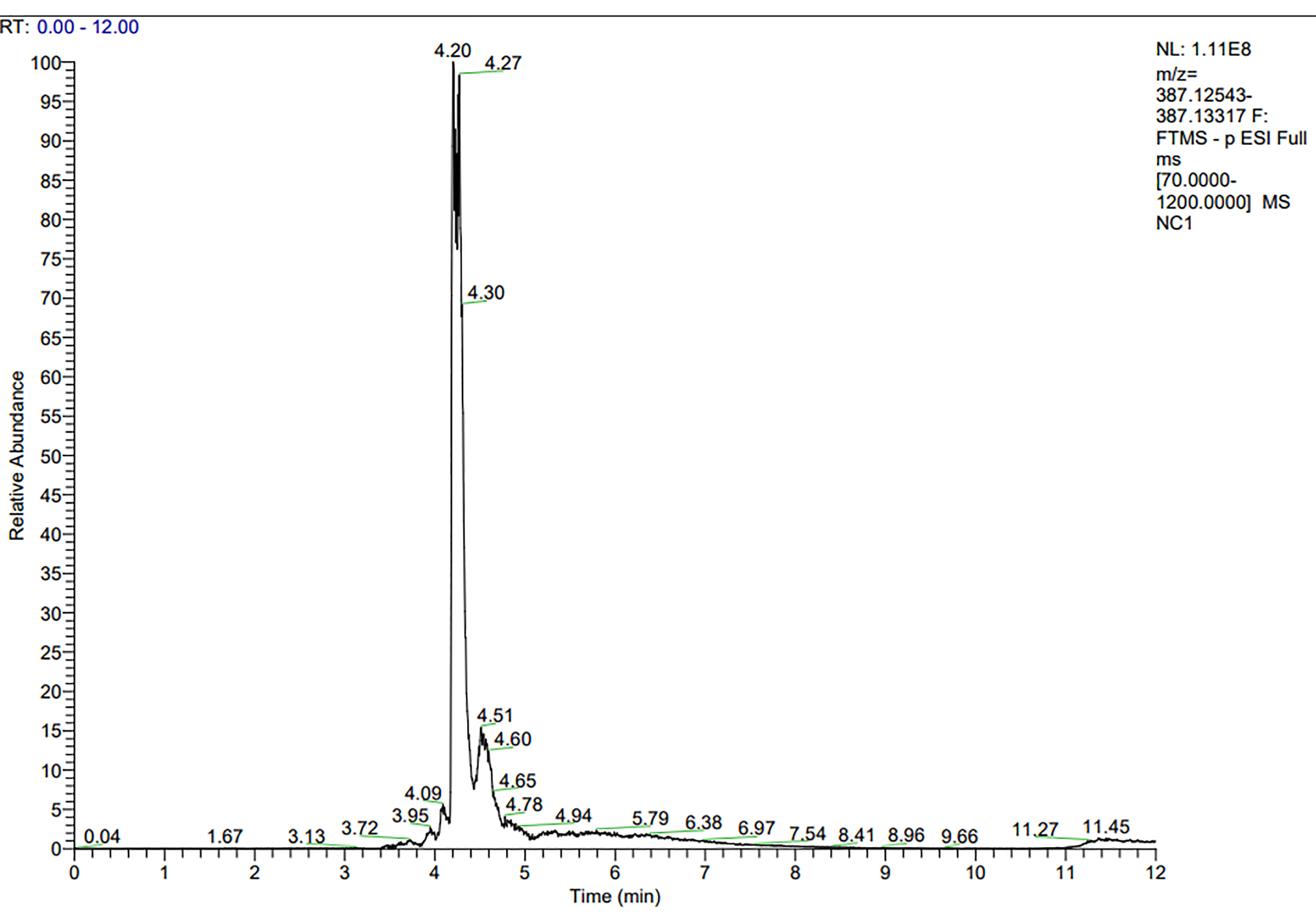

Supplement: Supplementary file 1 [file molecules-30-03764-s001.zip › Supplementary Figure S1/Geniposide.png]

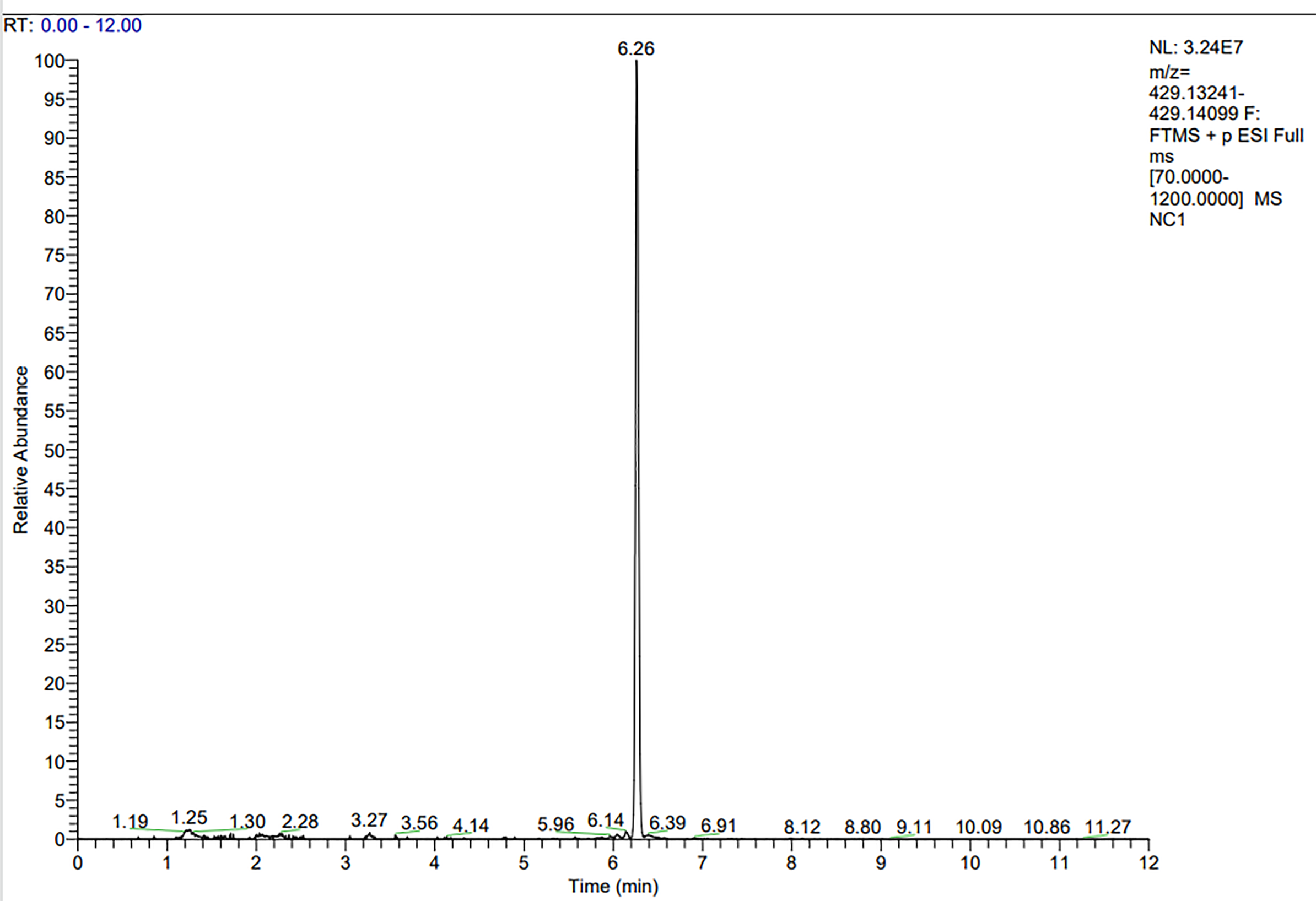

Supplement: Supplementary file 1 [file molecules-30-03764-s001.zip › Supplementary Figure S1/Shanzhiside methyl ester.png]

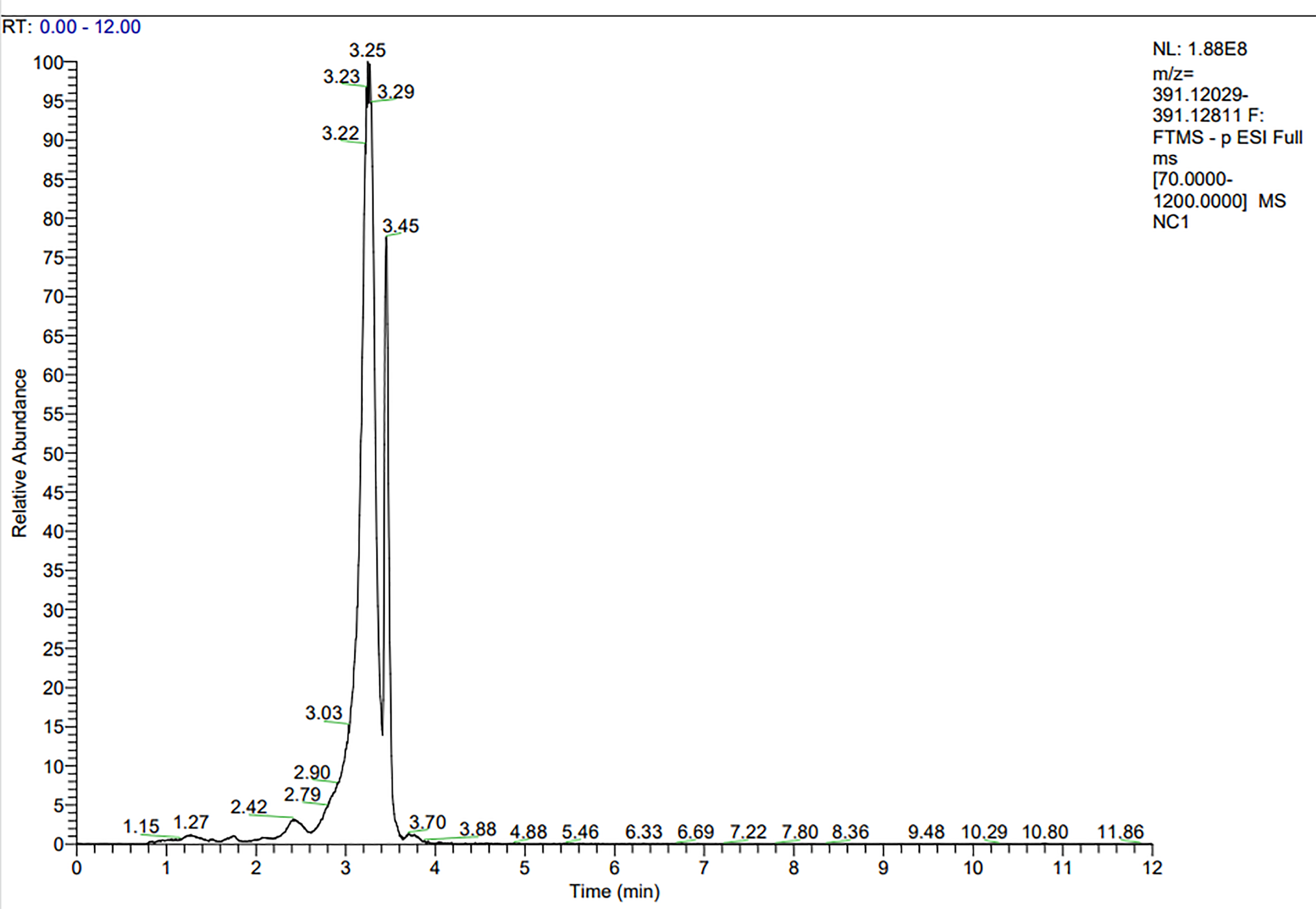

Supplement: Supplementary file 1 [file molecules-30-03764-s001.zip › Supplementary Figure S1/Shanzhiside.png]
